# Phonon-Induced Wake Potential in a Graphene–Insulator –Graphene Structure

**DOI:** 10.3390/nano14231951

**Published:** 2024-12-05

**Authors:** Ana Kalinić, Ivan Radović, Lazar Karbunar, Vito Despoja, Zoran L. Mišković

**Affiliations:** 1 Department of Atomic Physics, Vinča Institute of Nuclear Sciences—National Institute of the Republic of Serbia, University of Belgrade, P.O. Box 522, 11001 Belgrade, Serbia; ana.kalinic@vin.bg.ac.rs (A.K.); iradovic@vin.bg.ac.rs (I.R.); 2 School of Computing, Union University, Knez Mihailova 6, 11000 Belgrade, Serbia; lkarbunar@raf.rs; 3 Institute of Physics, Bijenička 46, 10000 Zagreb, Croatia; vdespoja@ifs.hr; 4 Department of Applied Mathematics, Waterloo Institute for Nanotechnology, University of Waterloo, Waterloo, ON N2L 3G1, Canada

**Keywords:** graphene, SiO_2_, HfO_2_, Al_2_O_3_, plasmon–phonon hybridization, wake potential

## Abstract

The aim of this study is to explore the potential which arises in a graphene–insulator–graphene structure when an external charged particle is moving parallel to it with a speed smaller than the Fermi speed in graphene. This is achieved by employing the dynamic polarization function of graphene within the random phase approximation, where its π electrons are modeled as Dirac fermions, and utilizing a local dielectric function for bulk insulators. Three different insulators are considered: SiO_2_, HfO_2_, and Al_2_O_3_. It is observed that the wake potential is induced by the surface optical phonons originating from the insulator layer, and that total potential could be effectively decomposed into two components, each corresponding to different phonon branches, as long as those branches do not interact amongst themselves.

## 1. Introduction

In the last twenty years, since the discovery of graphene in 2004 [1], two-dimensional (2D) materials have gathered significant scientific interest. Graphene, a monolayer of carbon atoms, features charge carriers known as massless Dirac fermions [2,3]. Doped graphene exhibits a low-frequency Dirac plasmon, along with the high-frequency π and σ + π plasmons [4]. The tunability of Dirac plasmon in the Terahertz (THz) to the mid-infrared (MIR) range of frequencies in graphene, which is facilitated by adjusting the charge carrier density via external gate(s), gives rise to applications in photonics, optoelectronics, transformation optics, plasmonics, and biosensing [5,6,7,8,9,10,11,12,13,14,15]. In experimental designs involving layered structures with graphene, the stacking of graphene sheets often includes insulating spacer layers [16,17,18]. Those layers typically support Fuchs–Kliewer (FK) or optical surface phonon modes which have the potential to attenuate Dirac plasmon or to hybridize with it. For example, it was observed [19,20] that graphene–insulator–graphene composite systems accommodate a range of intriguing plasmon–phonon hybrid modes across the THz to MIR frequency spectrum. On the other hand, the phononics of graphene, layered materials, and heterostructures has recently emerged as a compelling topic [21]. Particularly important for optical applications of phononics are the frequency regions where polar insulators exhibit the so-called Reststrahlen bands that extend between the transverse optical (TO) mode frequencies in the bulk of those materials and the corresponding FK frequencies.

The surface phonon modes can be efficiently probed by using slow charged particles under oblique incidence, such as in high-resolution electron energy loss spectroscopy (HREELS) [22] or in low-energy ion scattering (LEIS) [23]. Namely, when a charged particle moves parallel to a metallic or polar crystal surface, it loses its energy due to excitations of both individual particles and collective modes within that surface, which are accompanied by a re-distribution of the surface charge density. This phenomenon, known as the wake effect [24], is characterized by oscillatory potentials that emerge when the speed of the incoming particle matches the phase velocity of these collective oscillations. Consequently, the induced electrostatic potential forms a V-shaped wave pattern trailing behind the moving particle [25]. Research into the wake effect in 2D conductive materials began with theoretical studies of strongly coupled 2D electron gas [26] and has since expanded to include various graphene-based nanostructures [4,27,28,29,30]. In our earlier research [19,31,32,33], how plasmon–phonon hybridization affects the wake effect was examined, focusing on graphene supported by polar substrates. Those studies covered both individual [19,31,33] and correlated [32] charged particles traveling parallel to graphene. Also, it was found in the case of graphene–Al_2_O_3_–graphene composite system [4,30] that when the speed of the incident particle moving parallel to it is greater than the Fermi speed in graphene, the main contribution to the wake potential comes from the hybridized Dirac plasmons in the two graphene layers, whereas in the opposite case of a particle moving at a speed smaller than the Fermi speed, the TO phonon modes in the Al_2_O_3_ layer are the major contributors to the wake potential.

In this work we shall theoretically explore in greater detail the wake potential which occurs in the top layer of a graphene–insulator–graphene composite when the incident particle moves parallel to it with a speed smaller than the graphene’s Fermi speed. We focus on charged particles moving parallel to the surface of our structure, like in the case of ion grazing scattering [34] or aloof HREELS [35,36]. Three different insulators are considered: silicon dioxide (SiO_2_), hafnium dioxide (HfO_2_), and aluminum oxide (Al_2_O_3_), as they are often used in experiments as dielectric spacers [37,38,39,40,41]. All these oxides are known to exhibit several prominent TO phonon modes, which can all contribute to wake potential. In particular, if those modes are located at closely spaced frequencies, they may cause strong interference in the wake pattern. Here, we analyze the conditions under which the contributions of individual TO phonon modes, to the wake pattern, may be considered as decoupled from each other. This analysis can help elucidate the effects of the proximity of the corresponding Reststrahlen bands in polar insulators probed by HREELS or LEIS for phononics applications. The same geometry and parameters are used for all three insulators, being, namely, the speed and distance of the incident particle to the top graphene layer, the thickness of the insulator, the Fermi energy (i.e., the doping density) of graphene, and the damping rate of plasmons in graphene.

The following section will provide a brief overview of our theoretical model for an effective 2D dielectric function (and the corresponding loss function) and the wake potential. We shall then present and discuss our results. Finally, concluding remarks will be given in the last section.

Unless stated otherwise, Gaussian units of electrostatics will be used throughout this paper.

## 2. Theoretical Model

**Figure 1:**
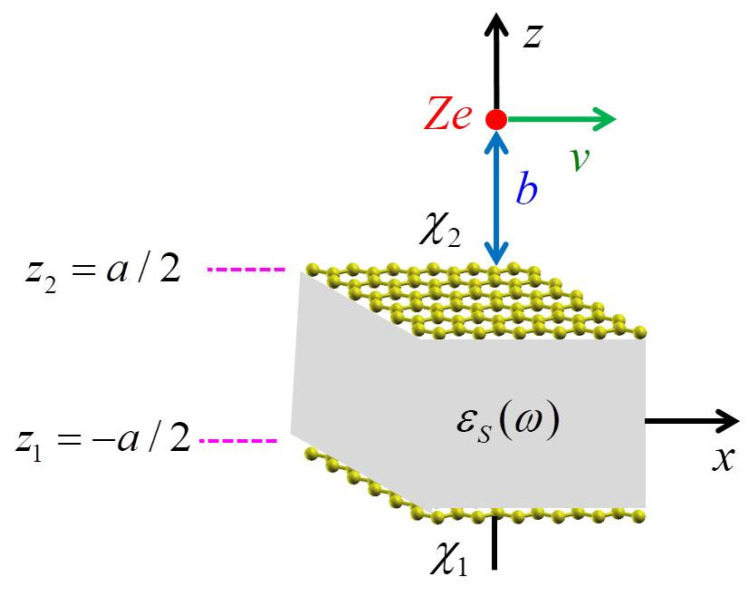
Illustration of the graphene–insulator–graphene setup featuring a point charge, Ze, moving parallel to the x-axis at a steady velocity v, positioned at a consistent distance b above the upper graphene layer. The polarization function of the top graphene layer situated in the $z_{2}=a/2$ plane is denoted as $\chi_{2},$ while that of the bottom layer at $z_{1}=-a/2$ is represented by $\chi_{1}.$ The dielectric layer with a thickness of a is characterized by its local dielectric function $\varepsilon_{s}(\omega )$.

A Cartesian coordinate system utilizing coordinates $\left\{\vec{R},z\right\}$ is employed, where $\vec{R}$ represents a 2D position vector $\vec{R}=\{x,y\}$ in the xy plane, and z signifies the distance from this plane. The composite structure of graphene–insulator–graphene, depicted in Figure 1, includes two graphene layers occupying the planes z=±a/2, with no gap between them and the insulator layer. The response functions of the bottom and top graphene sheets are denoted as $\chi_{1}$ and $\chi_{2}$, respectively, while each graphene sheet is presumed to be flat, has zero thickness, and is free from strain. The dielectric layer is regarded as a homogeneous isotropic slab characterized by its local dielectric function $\varepsilon_{s}(\omega ).$ The entire system is assumed to be situated in a vacuum or the air. In this scenario, it is assumed that a charged particle with the charge of Ze and velocity $\vec{v}$ is moving parallel to the composite along the x axis at a distance b from the upper graphene sheet. Therefore, its density can be expressed as $\rho_{ext}(\vec{R},z,t)=Ze\delta (\vec{R}-\vec{v}t)\delta [z-(a/2+b)].$ The total potential in the region above the upper graphene sheet, z≥a/2, may be written as [30]:

(1) $$\Phi_{tot}\left(\vec{R},z,t\right)=\frac{Ze}{{\left(2\pi \right)}^{2}}\int W\left(\vec{q},\vec{q}\cdot \vec{v},z,a/2+b\right)e^{i\vec{q}\cdot \left(\vec{R}-\vec{v}t\right)}d^{2}\vec{q}.$$

In our earlier publication [20], an expression for the screened Coulomb interaction $W(\vec{q},\omega ,z,z^{\prime })$, with $z,z^{\prime }\geq a/2$, was derived as:

(2) $$W\left(\vec{q},\omega ,z,z^{\prime }\right)=\frac{2\pi }{q}e^{-q\left|z-z^{\prime }\right|}+\frac{2\pi }{q}\left[\frac{1}{\varepsilon \left(\vec{q},\omega \right)}-1\right]e^{-q\left(z+z^{\prime }-a\right)},$$

where $\vec{q}=\{q_{x},q_{y}\}$ represents the momentum transfer vector parallel to the xy plane and $q=\sqrt{q_{x}^{2}+q_{y}^{2}}$. The effective 2D dielectric function $\varepsilon (\vec{q},\omega )$ can be expressed, in terms of the response functions of the non-interacting electrons in graphene layers and the local dielectric function of the insulator layer, as:

(3) $$\varepsilon \left(\vec{q},\omega \right)=\frac{1}{2}\left[1+\varepsilon_{s}\left(\omega \right)\coth\left(qa\right)+\frac{4\pi e^{2}}{q}\chi_{2}\left(q,\omega \right)\right]-\frac{1}{2}\frac{\varepsilon_{s}^{2}\left(\omega \right){\cos\mathrm{ech}}^{2}\left(qa\right)}{1+\varepsilon_{s}\left(\omega \right)\coth\left(qa\right)+\frac{4\pi e^{2}}{q}\chi_{1}\left(q,\omega \right)}.$$

Regarding the two response functions, $\chi_{1}$ and $\chi_{2}$, of each graphene sheet, we apply the massless Dirac fermion (MDF) model [42,43]. This method involves obtaining the density–density polarization functions for single-layer graphene with non-interacting electrons at zero temperature, using the random phase approximation. This approach considers only the π electron bands within the Dirac cone approximation. Damping effects are accounted for by using the Mermin procedure [30]. We assign equal doping across both graphene sheets, implying identical Fermi energies $E_{F}$ for each layer. This leads to equal polarization functions, $\chi_{1}=\chi_{2}\equiv \chi_{M}$, where the Mermin polarization function $\chi_{M}$ is defined by [30]:

(4) $$\chi_{M}\left(q,\omega ,\gamma \right)=\frac{\chi \left(q,\omega +i\gamma \right)}{1-\frac{i\gamma }{\omega +i\gamma }\left[1-\frac{\chi \left(q,\omega +i\gamma \right)}{\chi_{s}\left(q\right)}\right]},$$

where γ is the rate at which plasmons dampen, whereas the polarization χ(q,ω+iγ) and its static limit $\chi_{s}(q)=\underset{\omega ,\gamma \to 0}{\lim}\chi (q,\omega +i\gamma )$ are provided elsewhere [42,43].

Under the assumption that the polarization of the bulk insulator primarily arises from excitations of the two optical modes with the highest oscillator strengths [44,45], the bulk dielectric function of the oxides could be approximated as follows [46]:

(5) $$\varepsilon_{s}\left(\omega \right)=\varepsilon_{ox}^{\infty }+\left(\varepsilon_{ox}^{i}-\varepsilon_{ox}^{\infty }\right)\frac{\omega_{TO2}^{2}}{\omega_{TO2}^{2}-\omega^{2}-i\omega \gamma_{TO2}}+\left(\varepsilon_{ox}^{0}-\varepsilon_{ox}^{i}\right)\frac{\omega_{TO1}^{2}}{\omega_{TO1}^{2}-\omega^{2}-i\omega \gamma_{TO1}},$$

with $\varepsilon_{ox}^{\infty },$ $\varepsilon_{ox}^{i},$ and $\varepsilon_{ox}^{0}$ being the optical, intermediate, and static relative permittivities, $\omega_{TO1}$ and $\omega_{TO2}$ being the first and second TO angular frequencies $\left(\omega_{TO1}<\omega_{TO2}\right)$, and $\gamma_{TO1}$ and $\gamma_{TO2}$ being the damping rates of the TO phonons in question. Our goal is to elucidate the relative roles of the two TO modes in the wake formation. If the frequencies of those modes may be considered sufficiently separated, then it is possible to define two approximate forms of the above dielectric function. When only the first phonon is relevant $\left(\omega_{TO2}\to \infty \right)$, the dielectric function of the insulator is approximated by:

(6) $$\varepsilon_{s}^{\left(1\right)}\left(\omega \right)=\varepsilon_{ox}^{i}+\left(\varepsilon_{ox}^{0}-\varepsilon_{ox}^{i}\right)\frac{\omega_{TO1}^{2}}{\omega_{TO1}^{2}-\omega^{2}-i\omega \gamma_{TO1}},$$

whereas when only the second phonon is relevant $\left(\omega_{TO1}=0\right)$, we use the approximation:

(7) $$\varepsilon_{s}^{\left(2\right)}\left(\omega \right)=\varepsilon_{ox}^{\infty }+\left(\varepsilon_{ox}^{i}-\varepsilon_{ox}^{\infty }\right)\frac{\omega_{TO2}^{2}}{\omega_{TO2}^{2}-\omega^{2}-i\omega \gamma_{TO2}}.$$

In our calculations of the wake, besides using the full dielectric function of the insulator layer, we shall also test the presumed dominance of the lower-frequency and the higher-frequency TO modes by replacing the full dielectric function in Equation (5) with its approximate forms in Equation (6) and Equation (7), respectively.

Substituting Equation (2) into Equation (1), and assuming that a charged particle moves along the x-axis (as shown in Figure 1), an expression for the potential at the z=a/2 plane (i.e., the wake potential) is obtained as:

(8) Φtotx,y,z=a/2,t=2Zeπ∫0∞∫0∞e−qbqReeiqxx−vt+qyyεq,qxvdqxdqy,

where we have used the symmetry properties of the real and imaginary parts of the dielectric function ε(q,ω) from Equation (3). Note that the wake potential is stationary in the moving frame of reference attached to the external particle, allowing us to express the potential as $\Phi_{tot}\left(x,y,z=a/2,t\right)=\Phi_{tot}(x-vt,y,z=a/2).$

## 3. Results and Discussion

For the studied graphene–insulator–graphene composites it is assumed that both graphene sheets have the same Fermi energy, $E_{F}=200\;\mathrm{meV}$, and the same damping rate of plasmons, γ=10 meV. The selected thickness of the SiO_2_, HfO_2_, and Al_2_O_3_ layer is a=5 nm. The parameters used for the bulk dielectric function of the mentioned oxides, as given in Equation (5), are listed in Table 1 [44,45,46]. We set ℏ=1, thereby expressing all frequencies in units of energy. The external particle is chosen to be a proton (Z=1) moving with constant speed $v=0.5\;v_{F},$ where $v_{F}\approx 10^{6}\;m/s$ is the Fermi speed in graphene, at a distance b=0.5 nm above the upper graphene.

Firstly, we will discuss the loss function, given by Im[−1/ε(q,ω)], of the graphene–insulator–graphene composite systems for three different insulators. The effective 2D dielectric function in Equation (3), ε(q,ω), is obtained using the Mermin polarization function in Equation (4) and the dielectric function of the insulator in Equation (5). The dispersion relations for the collective modes in the observed systems are calculated by solving the equation ε(q,ω)=0. We set all damping rates in Equations (4) and (5) to zero, and use the optical limit (q≈0) of the MDF method for the polarization functions of the graphene layers (see paragraph F in Section II of Ref. [20]), to obtain the dispersion relations.

Then, we will discuss the wake potential in the graphene–insulator–graphene heterostructures for three different insulators.

The results regarding the loss functions were obtained using the MATLAB (MATrix LABoratory) software package (version R2023b), while the wake potentials were acquired using the FORTRAN (FORmula TRANslation) programming language.

### 3.1. The Loss Function

**Figure 2:**
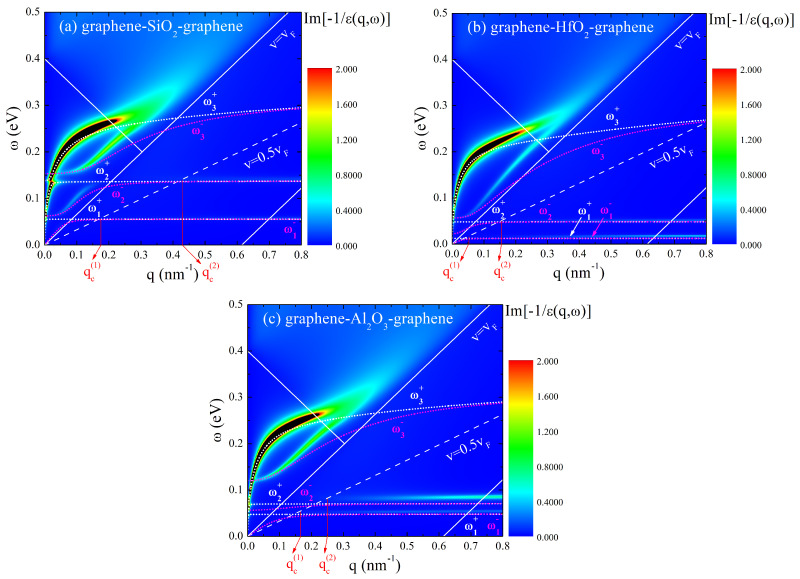
The loss function Im[−1/ε(q,ω)] (in arbitrary units) as a function of the wave number q (in nm^−1^) and the excitation frequency ω (in eV) for the graphene–insulator–graphene composite systems where the insulator is (**a**) SiO_2_, (**b**) HfO_2_, and (**c**) Al_2_O_3_. The magenta dotted lines depict the dispersion relations of three odd $\left(\omega_{i}^{-};\;i=1,2,3\right)$ plasmon–phonon modes, whereas the white dotted lines depict the dispersion relations of three even $\left(\omega_{i}^{+};\;i=1,2,3\right)$ plasmon–phonon modes. The white solid lines indicate the lower edge $\left(\omega =v_{F}(q-2k_{F})\right)$ and the upper edge $\left(\omega =v_{F}q\right)$ of the intraband π*↔π* electron–hole excitations, in addition to the lower edge $\left(\omega =2E_{F}-v_{F}q\right)$ of the interband π↔π* electron–hole excitations in the Dirac cone approximation. The white dashed lines represent the $\omega =0.5v_{F}q$ lines, helping to visualize their intersections (marked with $q_{c}^{\left(i\right)};\;i=1,2$) with eigenmodes that contribute to the wake potential.

Figure 2 shows the loss functions of the graphene–insulator–graphene composite systems where insulator is (a) SiO_2_, (b) HfO_2_, and (c) Al_2_O_3_. Knowing that each graphene sheet supports one Dirac plasmon and each insulator surface supports two FK phonons, the heterostructure supports six hybridized modes in total. The corresponding dispersion relations of the six plasmon–phonon hybridized modes (white and magenta dotted lines) are also shown in Figure 2. The white solid lines indicate the lower edge, $\omega =v_{F}(q-2k_{F}),$ and the upper edge, $\omega =v_{F}q,$ of the intraband π*↔π* electron–hole excitations, in addition to the lower edge, $\omega =2E_{F}-v_{F}q,$ of the interband π↔π* electron–hole excitations in the Dirac cone approximation. Here, $k_{F}=E_{F}/v_{F}=\sqrt{\pi |n|}$ represents graphene’s Fermi wave number, with n being the doping density.

The loss functions are dominated by the highest plasmon branches $(\omega_{3}^{\pm }),$ which result from the hybridization between Dirac plasmons in graphene layers. In the limit of long wavelengths (q→0), the lower branch $\left(\omega_{3}^{-}\right)$ shifts towards a finite frequency as a result of interacting with the polarization of the insulator layer. The peak positions of the loss functions agree well with the corresponding dispersion relations $\omega_{3}^{\pm }$ for $q<0.15{\;\mathrm{nm}}^{-1}$ in the cases of SiO_2_ (Figure 2a) and Al_2_O_3_ (Figure 2c) insulators, and for $q<0.1{\;\mathrm{nm}}^{-1}$ in the case of the HfO_2_ insulator (Figure 2b). It can also be seen that the peak positions of the loss functions for $\omega_{3}^{\pm }$ in all three cases merge at $q\approx 0.35{\;\mathrm{nm}}^{-1}$, whilst the corresponding dispersion’s curves are degenerate for much higher wave numbers, $q\approx 0.8{\;\mathrm{nm}}^{-1}$.

The other four branches $\left(\omega_{1,2}^{\pm }\right)$ are primarily derived from the surface phonon modes of the insulator. Each of the two phonon branch pairs become degenerate at larger q values, aligning with the bulk values of the first and second TO phonon frequencies given in Table 1. The loss functions in Figure 2 also exhibit two faint horizontal lines at still larger q values, which represent two FK phonon modes associated with the two corresponding TO modes [20]. Those lines occur at large wave numbers, so we may estimate their energies by setting qa>>1 (from which it follows that coth(qa)≈1 and cosech(qa)≈0) in Equation (3). In that case, Equation (3) becomes:

(9) $$\varepsilon \left(q,\omega \right)=\frac{1}{2}\left[1+\varepsilon_{s}\left(\omega \right)+\frac{4\pi e^{2}}{q}\chi_{2}\left(q,\omega \right)\right],$$

which then gives an effective dielectric function for a single graphene layer with polarization $\chi_{2}\left(q,\omega \right)$ on the surface of a semi-infinite substrate with the dielectric function $\varepsilon_{s}(\omega )$. Further, because we are interested in low speeds, we may approximate the polarization function of single-layer graphene by its static, short wavelength limit as $\chi_{s}=q/\left(4v_{F}\right)$ [20]. Thus, Equation (9) turns into:

(10) $$\varepsilon \left(\omega \right)\approx \frac{1}{2}\left[1+\varepsilon_{s}\left(\omega \right)+\frac{\pi e^{2}}{v_{F}}\right].$$

By setting all damping rates in Equation (5) to zero and solving the dispersion relation $1+\varepsilon_{s}\left(\omega \right)+\left(\pi e^{2}\right)/v_{F}=0,$ one obtains the screened FK phonon frequencies in the insulator substrates which are in close agreement with the positions of the two faint horizontal lines in the loss functions in Figure 2. The estimated FK phonon frequencies, along with the corresponding TO phonon frequencies, for SiO_2_, HfO_2_, and Al_2_O_3_ are given in Table 2. Notice that, in this regime, the primary role of graphene is to provide screening of the FK modes in the insulating layer, which is independent from the doping density of graphene, to the leading order in $\omega /v_{F}q$.

The white dashed lines in Figure 2 represent the $\omega =0.5v_{F}q$ lines, helping to visualize their intersections (marked with $q_{c}^{\left(i\right)};\;i=1,2$) with eigenmodes that contribute to the wake potential. One may see that the $v=0.5v_{F}$ lines cross only the FK phonon branches (i.e., they do not exceed any of the Dirac plasmonic branches), so that only the FK phonons in the insulating layers contribute to the wake potential. In the case of the SiO_2_ insulator, depicted in Figure 2a, the two faint horizontal lines in the loss function, which describe FK phonon modes, are already fully degenerated when the $v=0.5v_{F}$ line crosses them, resulting in the clearly defined values $q_{{c,\mathrm{SiO}}_{2}}^{\left(1\right)}=0.175{\;\mathrm{nm}}^{-1}$ and $q_{{c,\mathrm{SiO}}_{2}}^{\left(2\right)}=0.43{\;\mathrm{nm}}^{-1}.$ Also, they correspond excellently to the two dispersion curves, i.e., the $\omega_{1}^{\pm }$ and $\omega_{2}^{\pm }$ lines, representing the TO surface phonon modes. For the HfO_2_ insulator, shown in Figure 2b, the FK branches and the corresponding TO lines are slightly shifted relative to each other, but are still both almost fully degenerated when the $v=0.5v_{F}$ line intersect with them. The wavenumber values at which that happens are $q_{{c,\mathrm{HfO}}_{2}}^{\left(1\right)}=0.052{\;\mathrm{nm}}^{-1}$ and $q_{{c,\mathrm{HfO}}_{2}}^{\left(2\right)}=0.155{\;\mathrm{nm}}^{-1}.$ Lastly, when Al_2_O_3_ is the insulator, as in Figure 2c, the situation is somewhat complicated. The phonon modes are much closer to each other than in the previous cases, the dispersion curves $\omega_{1,2}^{\pm }$ are not degenerated when the $v=0.5v_{F}$ line crosses them, and there is a visible vertical shift between the FK branches and the corresponding TO lines. In addition, the branches themselves are thicker, and the wavenumbers have the values of $q_{{c,\mathrm{Al}}_{2}O_{3}}^{\left(1\right)}=0.168{\;\mathrm{nm}}^{-1}$ and $q_{{c,\mathrm{Al}}_{2}O_{3}}^{\left(2\right)}=0.25{\;\mathrm{nm}}^{-1}$.

The critical wave numbers $q_{c}^{\left(i\right)}$ (i=1,2) in all three cases are summarized in Table 3.

### 3.2. The Wake Potential

**Figure 3:**
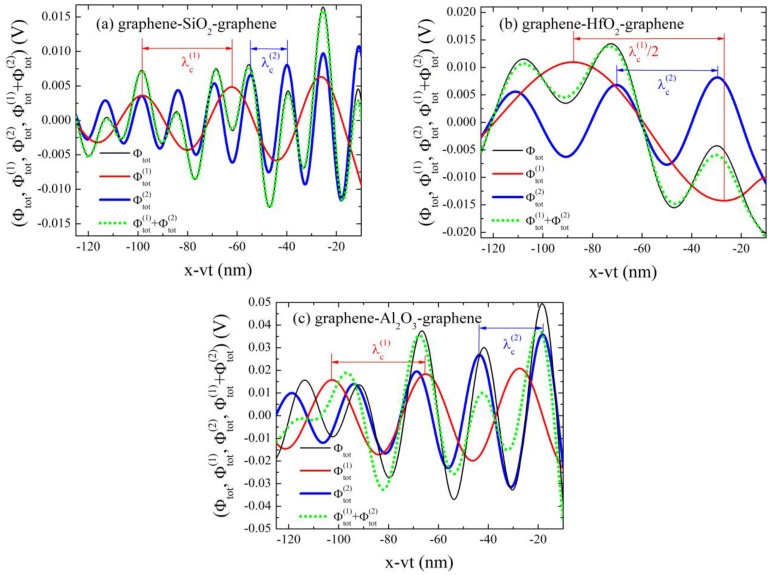
The wake potential (in V) as a function of the x−vt (in nm) with y=0 in the graphene–insulator–graphene composite systems where the insulator is (**a**) SiO_2_, (**b**) HfO_2_, and (**c**) Al_2_O_3_. The thin black lines show total potential $\left(\Phi_{tot}\right)$, the medium red lines show potential when only the first phonon is observed $\left(\Phi_{tot}^{\left(1\right)}\right)$, the thick blue lines show potential when only the second phonon is present $\left(\Phi_{tot}^{\left(2\right)}\right)$, and the green dotted lines are the sum of the red and the blue ones $\left(\Phi_{tot}^{\left(1\right)}+\Phi_{tot}^{\left(2\right)}\right)$. The characteristic wavelengths $\left(\lambda_{c}^{\left(i\right)};\;i=1,2\right)$ are highlighted.

Figure 3 shows the total wake potential $\Phi_{tot}$ (thin black lines), the “partial” potential $\Phi_{tot}^{\left(1\right)}$ when only the first phonon is observed (medium red lines), the “partial” potential $\Phi_{tot}^{\left(2\right)}$ when only the second phonon is present (thick blue lines), and the potential $\Phi_{tot}^{\left(1\right)}+\Phi_{tot}^{\left(2\right)}$ which is the sum of the two “partial” potentials (green dotted lines), in the graphene–insulator–graphene composite systems where insulator is (a) SiO_2_, (b) HfO_2_, and (c) Al_2_O_3_. The potentials are plotted as functions of x−vt for y=0 in the z=a/2 plane. The total potential $\Phi_{tot}$ was calculated using Equation (8) in combination with Equation (5), while the “partial” potentials $\Phi_{tot}^{\left(1\right)}$ and $\Phi_{tot}^{\left(2\right)}$ were obtained using Equation (8) in combination with Equation (6) and Equation (7), respectively. The characteristic wavelength $\lambda_{c}^{\left(i\right)}$ of oscillations in the potential when only the first or second phonon are included can be established using a critical wave number $q_{c}^{\left(i\right)}$, at which the $\omega =0.5v_{F}q$ line intersects the corresponding FK phonon branch in the loss function:

(11) $$\lambda_{c}^{\left(i\right)}=\frac{2\pi }{q_{c}^{\left(i\right)}};\;i=1,2.$$

In the case of the SiO_2_ insulator, the characteristic wavelengths of the potentials $\Phi_{{\mathrm{tot},\mathrm{SiO}}_{2}}^{\left(1\right)}$ and $\Phi_{{\mathrm{tot},\mathrm{SiO}}_{2}}^{\left(2\right)}$, determined directly from Figure 3a, are $\lambda_{{c,\mathrm{SiO}}_{2}}^{\left(1\right)}\approx 36\;\mathrm{nm}$ and $\lambda_{{c,\mathrm{SiO}}_{2}}^{\left(2\right)}\approx 14.5\;\mathrm{nm}$, respectively, which are in excellent agreement with the wavelengths calculated from Equation (11) using $q_{{c,\mathrm{SiO}}_{2}}^{\left(1\right)}=0.175{\;\mathrm{nm}}^{-1}$ and $q_{{c,\mathrm{SiO}}_{2}}^{\left(2\right)}=0.43{\;\mathrm{nm}}^{-1}$ from Figure 2a. For the HfO_2_ insulator, the characteristic wavelengths from Figure 3b of the $\Phi_{{\mathrm{tot},\mathrm{HfO}}_{2}}^{\left(1\right)}$ and $\Phi_{{\mathrm{tot},\mathrm{HfO}}_{2}}^{\left(2\right)}$ are $\lambda_{{c,\mathrm{HfO}}_{2}}^{\left(1\right)}\approx 121\;\mathrm{nm}$ and $\lambda_{{c,\mathrm{HfO}}_{2}}^{\left(2\right)}\approx 40.5\;\mathrm{nm}$, respectively, which perfectly match the calculated wavelengths from Equation (11) using $q_{{c,\mathrm{HfO}}_{2}}^{\left(1\right)}=0.052{\;\mathrm{nm}}^{-1}$ and $q_{{c,\mathrm{HfO}}_{2}}^{\left(2\right)}=0.155{\;\mathrm{nm}}^{-1}$ from Figure 2b. When Al_2_O_3_ is the insulator, as in Figure 3c, the determined characteristic wavelengths of the $\Phi_{{\mathrm{tot},\mathrm{Al}}_{2}O_{3}}^{\left(1\right)}$ and $\Phi_{{\mathrm{tot},\mathrm{Al}}_{2}O_{3}}^{\left(2\right)}$ are $\lambda_{{c,\mathrm{Al}}_{2}O_{3}}^{\left(1\right)}\approx 37.5\;\mathrm{nm}$ and $\lambda_{{c,\mathrm{Al}}_{2}O_{3}}^{\left(2\right)}\approx 25\;\mathrm{nm}$, respectively, which correspond excellently to the calculated wavelengths from Equation (11) using $q_{{c,\mathrm{Al}}_{2}O_{3}}^{\left(1\right)}=0.168{\;\mathrm{nm}}^{-1}$ and $q_{{c,\mathrm{Al}}_{2}O_{3}}^{\left(2\right)}=0.25{\;\mathrm{nm}}^{-1}$ from Figure 2c. This proves that, in the cases when only the first or second phonons are included, the wake potential comes from excitations of the corresponding FK phonons. In all three cases, the amplitude of the potentials is dependent on the intensity of the FK phonon branches in the loss functions, with Al_2_O_3_ phonons being the strongest.

The characteristic wavelengths $\lambda_{c}^{\left(i\right)}$ (i=1,2) in all three cases are summarized in Table 4.

In Figure 3a, in the case of the SiO_2_ insulator, it can be seen that the green dotted line matches perfectly with the thin black line, meaning that the total potential $\Phi_{tot}$ could be perceived as the superposition of the $\Phi_{tot}^{\left(1\right)}$ and $\Phi_{tot}^{\left(2\right)}$ potentials. As can be seen in Figure 3b, for the HfO_2_ insulator, the sum of the potentials $\Phi_{tot}^{\left(1\right)}$ and $\Phi_{tot}^{\left(2\right)}$ agrees very well with the total potential $\Phi_{tot}$. In contrast, it is clear from Figure 3c that in the case of the Al_2_O_3_ insulator, the green dotted line does not agree well with the thin black line, suggesting that the total wake potential $\Phi_{tot}$ is not a mere summation of the corresponding $\Phi_{tot}^{\left(1\right)}$ and $\Phi_{tot}^{\left(2\right)}$ potentials but rather exhibits significant interference effects. A possible explanation for the discrepancy of the total potential $\Phi_{tot}$ and the sum of the potentials $\Phi_{tot}^{\left(1\right)}$ and $\Phi_{tot}^{\left(2\right)}$ may come from the fact that in the graphene–Al_2_O_3_–graphene composite system the FK phonon frequencies are close to each other, so the two modes interact strongly. This is discussed further in the Supplementary Materials (SM).

## 4. Conclusions

We have explored the phonon excitations in a graphene–insulator–graphene composite system due to an external charged particle moving parallel to it with the velocity less than the Fermi velocity in graphene. The resulting wake potential (the total potential in the plane of the upper graphene sheet) was calculated using the polarization function of graphene’s π electrons in the random phase approximation and using a local dielectric function for the insulator. We have considered three different insulators: silicon dioxide (SiO_2_), hafnium dioxide (HfO_2_), and aluminum oxide (Al_2_O_3_). It was shown that in all three cases only the surface optical phonons from the insulator layer contribute to the wake potential. When only the first or second phonons are included, the wake potential comes from the excitations of the corresponding FK phonons and the amplitude of the potentials is dependent on their intensities in the loss functions. When the insulator is SiO_2_ or HfO_2_, the total potential can be nicely decomposed into two parts originating from the two FK phonon modes without any interferences between them. However, when the insulator is Al_2_O_3_, that is not the case. There are differences between the total potential and the sum of individual potentials, indicating strong interference effects. This is likely due to the fact that the relevant FK phonon frequencies are close to each other, so the interaction between the two FK modes in Al_2_O_3_ becomes strong.

Different characteristics and origins of the wake pattern discussed in this paper can provide theoretical insights into plasmon–phonon hybridization in aforementioned structures. It should be mentioned that, while the role of graphene in the above analysis is to merely screen the FK modes, its presence on the surface of the oxide layers can be useful in experiments that require smooth surfaces. The geometry of the analyzed systems, i.e., when the wake effect is caused by a charged particle traveling parallel to a solid surface or a 2D material on a substrate, could be readily achieved in the HREELS and LEIS experimental probes. This may be further relevant in other surface-sensitive techniques, such as low-energy ion-surface grazing scattering [47] or in the context of aloof trajectories of electron beams utilized for vibrational and valence EELS in scanning transmission electron microscopy [48].

## Figures and Tables

**Table 1:** Parameters used for the bulk dielectric functions of SiO_2_, HfO_2_, and Al_2_O_3_ [44,45,46].

	SiO_2_	HfO_2_	Al_2_O_3_
$\varepsilon_{ox}^{0}$	3.90	22.00	12.53
$\varepsilon_{ox}^{i}$	3.05	6.58	7.27
$\varepsilon_{ox}^{\infty }$	2.50	5.03	3.20
$\omega_{TO1}\;[\mathrm{meV}]$	55.60	12.40	48.18
$\omega_{TO2}\;[\mathrm{meV}]$	138.10	48.35	71.41
$\gamma_{TO1}\;[\mathrm{meV}]$	5.37	2.32	1.74
$\gamma_{TO2}\;[\mathrm{meV}]$	8.95	3.10	6.82

**Table 2:** TO and FK phonon frequencies for SiO_2_, HfO_2_, and Al_2_O_3_.

	SiO_2_	HfO_2_	Al_2_O_3_
$\omega_{TO1}\;[\mathrm{meV}]$	55.60	12.40	48.18
$\omega_{TO2}\;[\mathrm{meV}]$	138.10	48.35	71.41
$\omega_{FK1}\;[\mathrm{meV}]$	57.70	17.76	54.00
$\omega_{FK2}\;[\mathrm{meV}]$	141.80	51.40	86.53

**Table 3:** Critical wave numbers $q_{c}^{\left(i\right)}$ (i=1,2) from Figure 2 for graphene–insulator–graphene composite systems where insulator is (a) SiO_2_, (b) HfO_2_, and (c) Al_2_O_3_.

	(a) SiO_2_	(b) HfO_2_	(c) Al_2_O_3_
$q_{c}^{\left(1\right)}\;[{\mathrm{nm}}^{-1}]$	0.175	0.052	0.168
$q_{c}^{\left(2\right)}\;[{\mathrm{nm}}^{-1}]$	0.43	0.155	0.25

**Table 4:** Characteristic wavelengths $\lambda_{c}^{\left(i\right)}$ (i=1,2) from Figure 3 for graphene–insulator–graphene composite systems where the insulator is (a) SiO_2_, (b) HfO_2_, and (c) Al_2_O_3_.

	(a) SiO_2_	(b) HfO_2_	(c) Al_2_O_3_
$\lambda_{c}^{\left(1\right)}\;[\mathrm{nm}]$	36	121	37.5
$\lambda_{c}^{\left(2\right)}\;[\mathrm{nm}]$	14.5	40.5	25

## Data Availability

The data presented in this study are available on request from the corresponding author.

## Supplementary Materials

The following supporting information can be downloaded at: https://www.mdpi.com/article/10.3390/nano14231951/s1: Figure S1. The loss function Im[−1/ε(q,ω)] (in arbitrary units) as a function of the wave number q (in nm^−1^) and the excitation frequency ω (in eV) for the graphene-insulator-graphene composite systems where insulator is (a) SiO_2_, (b) HfO_2_, and (c) Al_2_O_3_, when only the first phonon is observed (panels a1, b1, and c1), when only the second phonon is presented (panels a2, b2, and c2), and when both phonons are included (panels a3, b3, and c3). The white solid lines indicate the lower edge $\left(\omega =v_{F}(q-2k_{F})\right)$ and the upper edge $\left(\omega =v_{F}q\right)$ of the intraband π*↔π* electron-hole excitations, in addition to the lower edge $\left(\omega =2E_{F}-v_{F}q\right)$ of the interband π↔π* electron-hole excitations in the Dirac cone approximation. The white dashed lines represent the $\omega =0.5v_{F}q$ lines.
