# Supplementary material for: Phonon-Induced Wake Potential in a Graphene–Insulator –Graphene Structure
Source: Nanomaterials (Basel). 2024 Dec 5;14(23):1951. doi: 10.3390/nano14231951 (PMC11643178; doi:10.3390/nano14231951)
Supplement: Supplementary file 1 [file nanomaterials-14-01951-s001.zip › nanomaterials-3290376-supplementary.pdf]

Supporting Information

In Figure S1 we compare the loss functions for the graphene-insulator-graphene composite systems where insulator is (a) SiO_2, (b) HfO_2, and (c) Al_2O_3, in the cases when only the first phonon is observed (panels a1, b1, and c1), when only the second phonon is presented (panels a2, b2, and c2), and when both phonons are included (panels a3, b3, and c3). It is evident that, when both phonons are included, in the cases of SiO_2 (panel a3) and HfO_2 (panel b3) insulators, the intensities of the two Fuchs-Kliewer (FK) phonon branches are the same as in the cases when only the first (panels a1 and b1) or second (panels a2 and b2) phonons are included. This suggests that in the cases of SiO_2 and HfO_2 insulators the two FK phonon modes do not interact with each other. As a result, the total potential could be nicely decomposed into two parts originating from those modes without any interference between them (as shown in Figure 3a and Figure 3b in the main text). However, in the case of Al_2O_3 insulator, the intensities of the two FK phonon branches (panel c3) are significantly changed in comparison with the cases when only the first (panel c1) or second (panel c2) phonons are included, indicating a strong spectral weight redistribution for interacting phonons. It can be concluded that in this case the two FK modes interact strongly, which causes the differences between the total potential and the sum of individual potentials (as shown in Figure 3c in the main text).

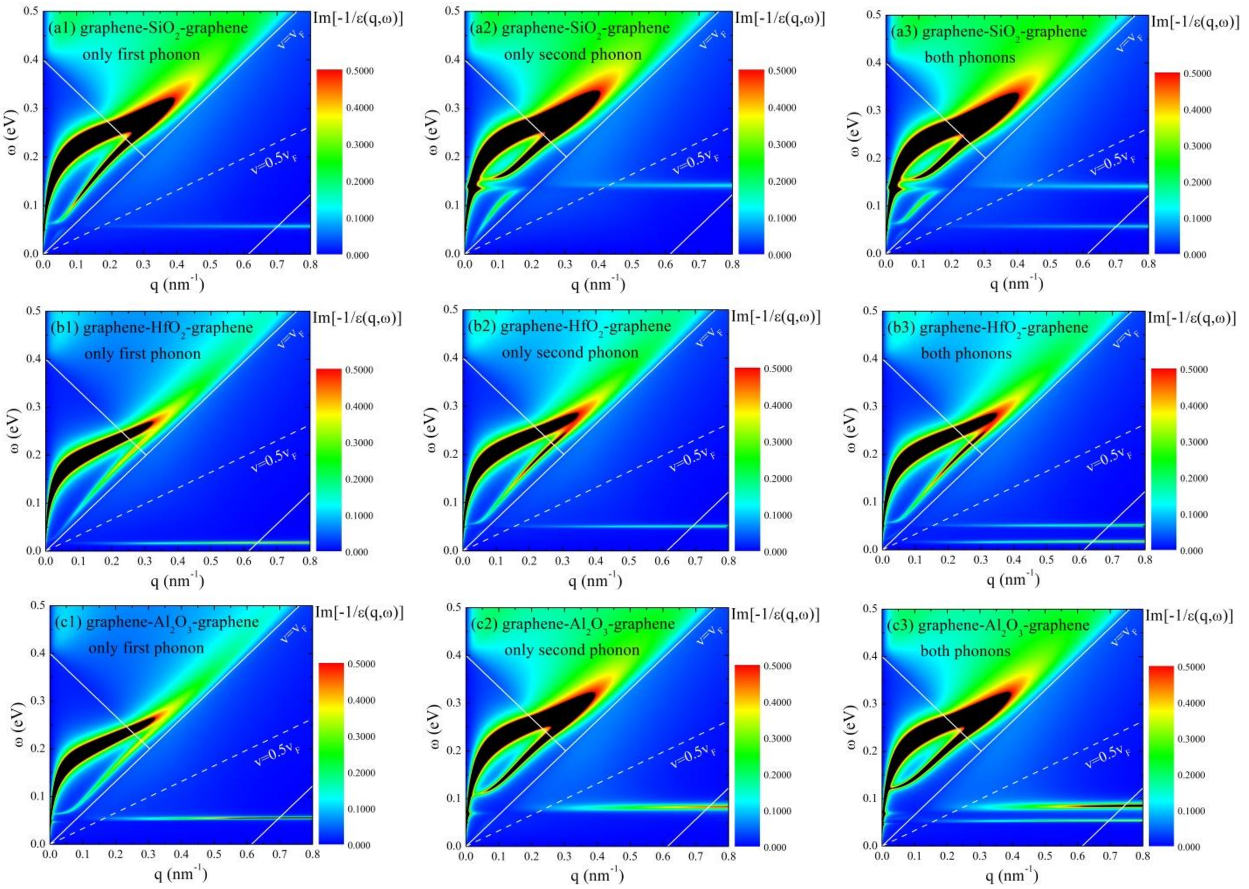

Figure S1. The loss function $\mathrm{Im}[-1/\varepsilon(q,\omega)]$ (in arbitrary units) as a function of the wave number q (in nm^{-1}) and the excitation frequency ω (in eV) for the graphene-insulator-graphene composite systems where insulator is (a) SiO_2, (b) HfO_2, and (c) Al_2O_3, when only the first phonon is observed (panels a1, b1, and c1), when only the second phonon is presented (panels a2, b2, and c2), and when both phonons are included (panels a3, b3, and c3). The white solid lines indicate the lower edge $(\omega = v_F(q-2k_F))$ and the upper edge $(\omega = v_F q)$ of the intraband $\pi^* \leftrightarrow \pi^*$ electron-hole excitations, in addition to the lower edge $(\omega = 2E_F - v_F q)$ of the interband $\pi \leftrightarrow \pi^*$ electron-hole excitations in the Dirac cone approximation. The white dashed lines represent the $\omega = 0.5 v_F q$ lines.
